# Supplementary material for: SOX-5 activates a novel RORγt enhancer to facilitate experimental autoimmune encephalomyelitis by promoting Th17 cell differentiation
Source: Nat Commun. 2021 Jan 20;12:481. doi: 10.1038/s41467-020-20786-w (PMC7817841; doi:10.1038/s41467-020-20786-w)
Supplement: Supplementary file 1 — Supplementary Information [file 41467_2020_20786_MOESM1_ESM.pdf]

SUPPLEMENTARY INFORMATION

SOX-5 activates a novel RORγt enhancer to facilitate experimental autoimmune encephalomyelitis by promoting Th17 cell differentiation

Yi Tian, Chao Han, Zhiyuan Wei, Hui Dong, Xiaohe Shen, Yiqiang Cui, Xiaolan Fu, Zhiqiang Tian, Shufeng Wang, Jian Zhou, Di Yang, Yi Sun, Jizhao Yuan, Bing Ni, Yuzhang Wu

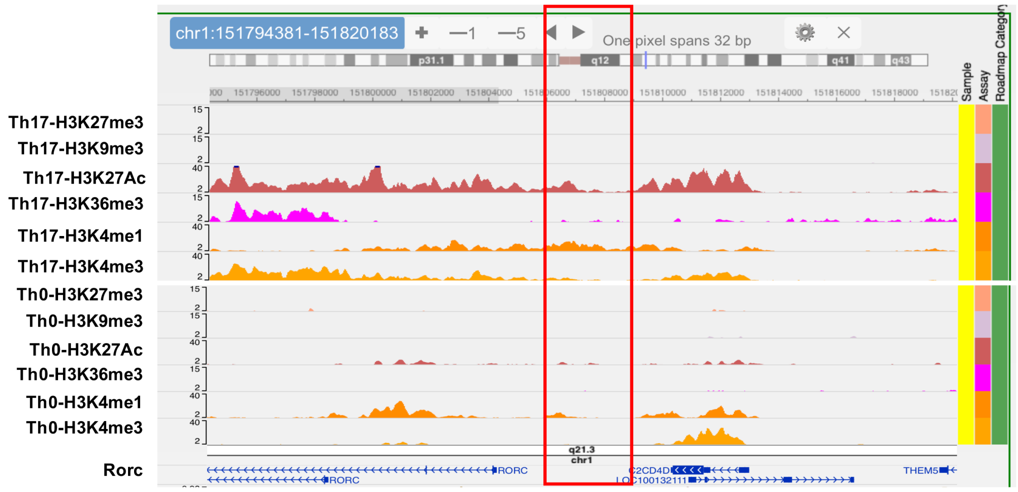

Supplementary Figure 1. Chromatin signature analysis of a region in human Th17 and Th0 cells similar to mouse RORCE.

Chromatin signature analysis of a region similar to mouse RORCE (indicated by the red box) including H3K27me3, H3K9me3, H3K27Ac, H3K36me3, H3K4me1 and H3K4me3 in human Th17 and Th0 cells with the Roadmap Epigenomics Project database (http://egg2.wustl.edu/roadmap/web_portal/).

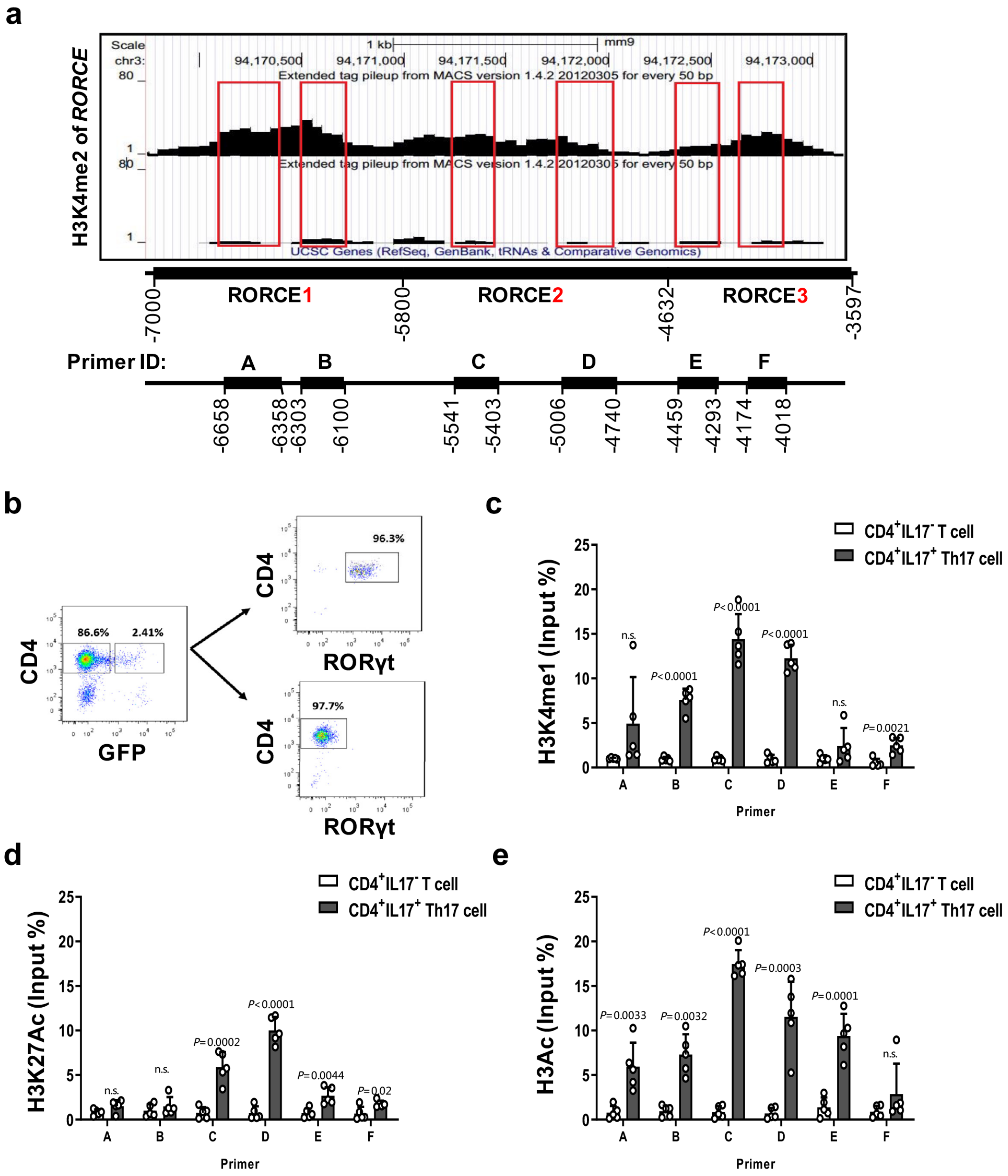

Supplementary Figure 2. Validation of active enhancer-associated epigenetic markers in RORCE in Th17 cells.

a. Based on the median size of common enhancers, RORCE was subdivided into three consecutive fragments: RORCE1 (chr3: 94,169,746-94,170,945), RORCE2 (chr3: 94,170,946-94,172,113), and RORCE3 (chr3: 94,172,215-94,173,149). Red boxes represent the targeted regions detected by ChIP-qPCR with 6 corresponding primer pairs (A-F). b. Sorting of Th17 and non-Th17 cells from IL-17A-EGFP reporter mice was performed by FACS. Splenic $CD4^+$ T cells were sorted into $CD4^+GFP^+$ Th17 and $CD4^+GFP^-$ non-Th17 cells, and the purities of sorted cells were further confirmed with an anti-RORγt mAb before use. c-e. ChIP-qPCR analysis for H3K4me1 (c), H3K27Ac (d) and H3Ac (e) in the RORCE region of the sorted cells is shown. Mean ± SEM are shown, n=5 independent experiments, unpaired two-tailed Student's t-test (c-e). Experimental mice were between 8 and 12 weeks of age, with no preference to gender and were maintained on a C57BL/6 background. Source data are provided as a Source Data file.

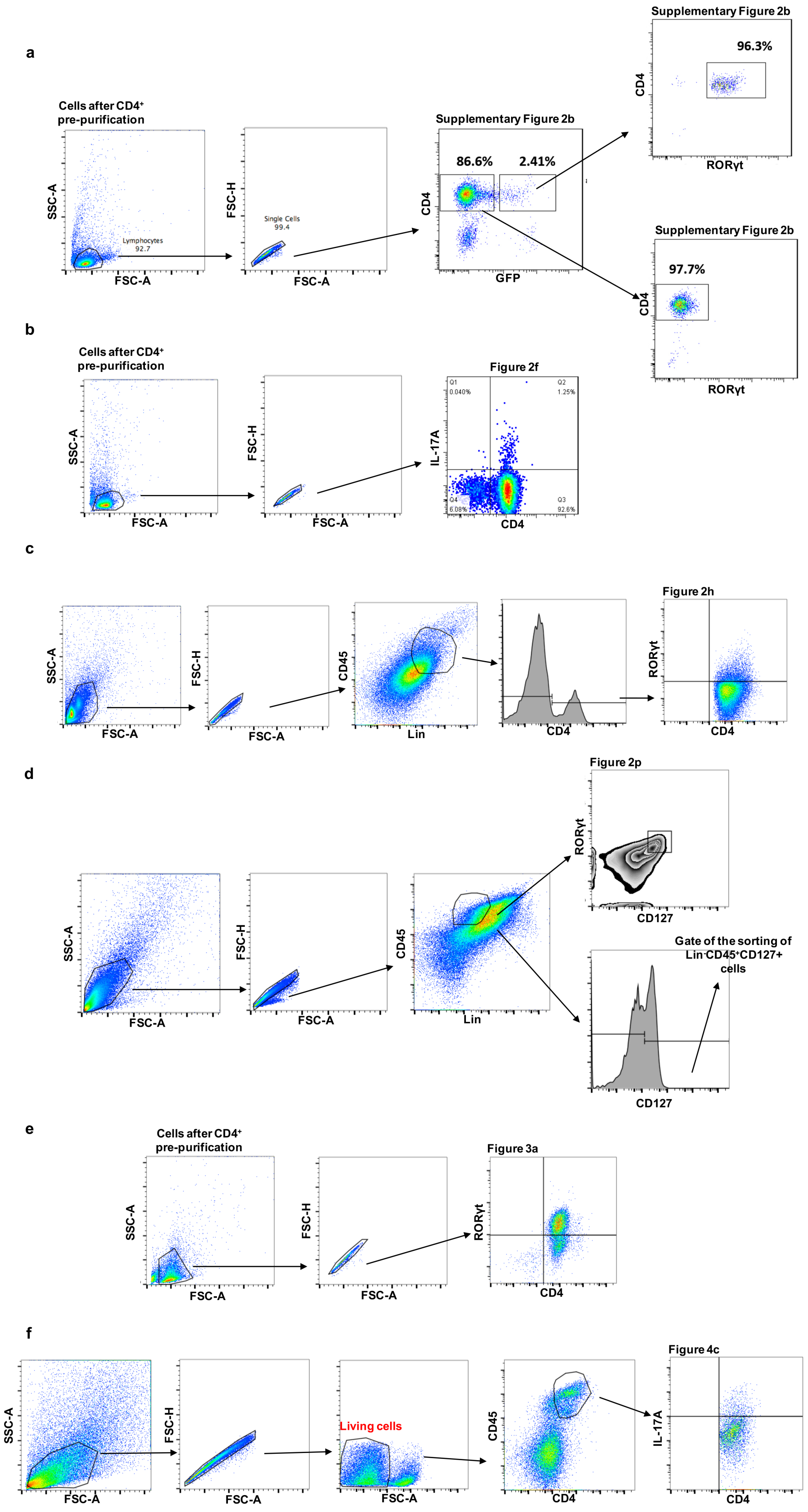

a
Cells after CD4+ pre-purification
SSC-A
FSC-A
Lymphocytes 92.7
FSC-H
Single Cells 99.4
Supplementary Figure 2b
CD4
GFP
86.6%
2.41%
Supplementary Figure 2b
96.3%
CD4
RORγt
Supplementary Figure 2b
97.7%
CD4
RORγt
b
Cells after CD4+ pre-purification
SSC-A
FSC-A
FSC-H
FSC-A
Figure 2f
IL-17A
CD4
Q1 0.040%
Q2 1.25%
Q4 6.08%
Q3 92.6%
c
SSC-A
FSC-A
FSC-H
FSC-A
CD45
Lin
CD4
Figure 2h
RORγt
CD4
d
SSC-A
FSC-A
FSC-H
FSC-A
CD45
Lin
Figure 2p
RORγt
CD127
Gate of the sorting of Lin-CD45+CD127+ cells
CD127
e
Cells after CD4+ pre-purification
SSC-A
FSC-A
FSC-H
FSC-A
Figure 3a
RORγt
CD4
f
SSC-A
FSC-A
FSC-H
FSC-A
Living cells
FSC-A
CD45
CD4
Figure 4c
IL-17A
CD4

Supplementary Figure 3. Gating strategies used for cell sorting and frequency analysis.

a. Gating strategy to sort Th17 ($CD4^+GFP^+$) and non-Th17 cells ($CD4^+GFP^-$) from IL-17A-EGFP reporter mice presented on supplementary Figure 2. b. Gating strategy to determine the percentage of $CD4^+$IL-$17A^+$Th17 cells in splenic $CD4^+$ T cells presented on Figure 2f and the same strategy was used in Figure 2c, supplementary Figure 4b, supplementary Figure 4d, Figure 7b, Figure 7d. c. Gating strategy to determine the percentage of RORγt^+ Th17 in $CD4^+CD45^+$ Lin^+ lymphocyte population from LP of small intestine presented on Figure 2h and the same strategy was used in Figure 2l, Figure 7f, Figure 7h. d. Gating strategy to determine the percentage of $CD127^+$ RORγt^+ ILC3 cells in the $CD45^+$ Lin^- lymphocyte population of the lamina propria presented on Figure 2p. e. Gating strategy to determine the percentage of $CD4^+$RORγt^+ Th17 cells that differentiated from Naïve $CD4^+$ T cells presented on Figure 3a and the same strategy was used in Figure 3d, Figure 3i, Figure 3m, Figure 7i, Figure 7j. f. Gating strategy to analyze the percentage of $CD4^+$IL-$17A^+$Th17 cells in $CD45^+CD4^+$ T cells of the spinal cord of indicated mice presented on Figure 4c and the same strategy was used in Figure 4d, Figure 4e, Figure 8c.

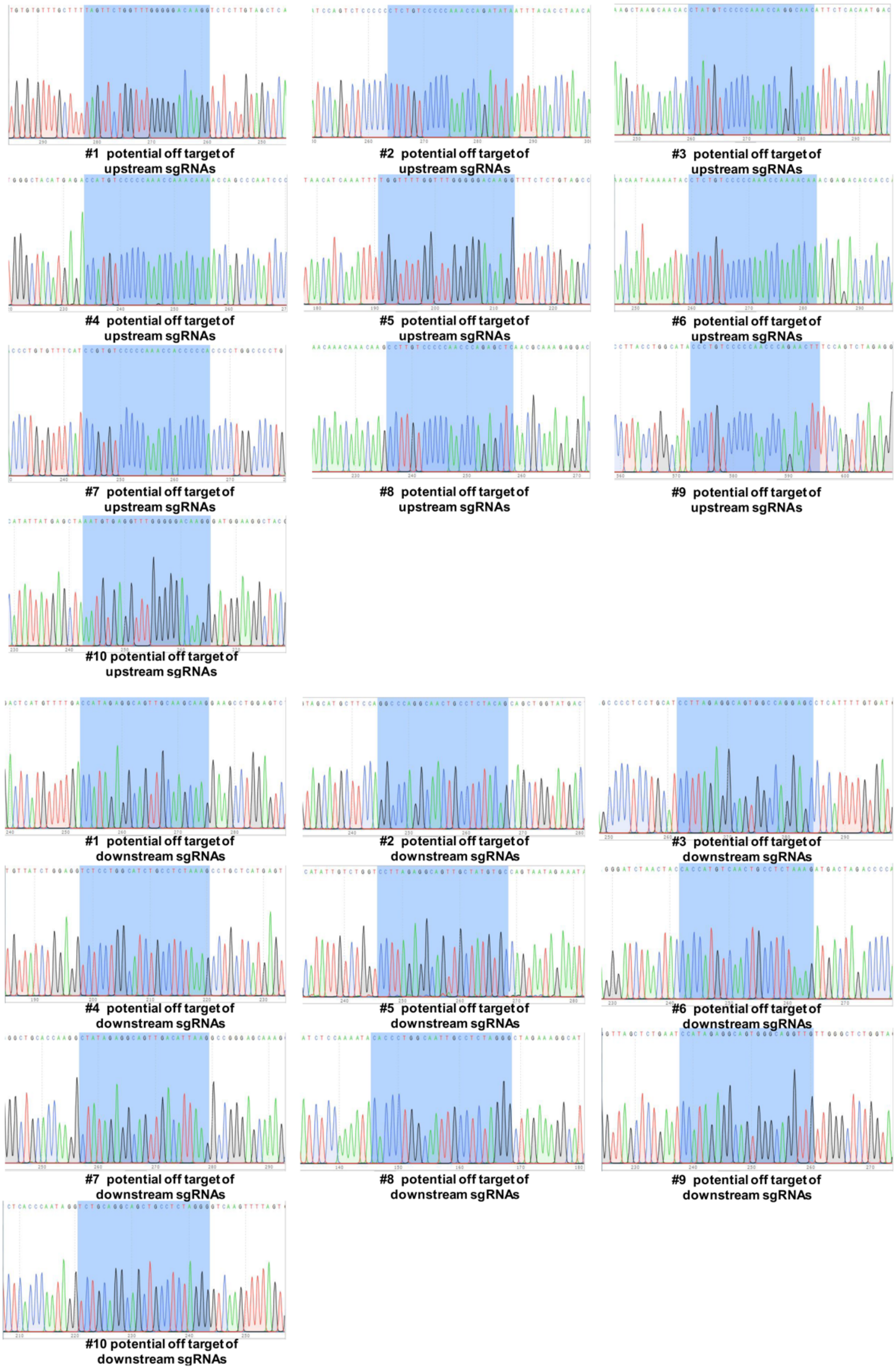

Supplementary Figure 4. Sequencing results of top 10 potential off targets of upstream sgRNAs or downstream sgRNAs used in RORCE2$^{-/-}$mice

Top 10 potential off targets of upstream sgRNAs or downstream sgRNAs used in RORCE2$^{-/-}$mice were amplified by PCR with genomic DNA of RORCE2$^{-/-}$mice and then PCR products were sequenced. PCR primers used for this assay are listed in Supplementary **Table 1.**

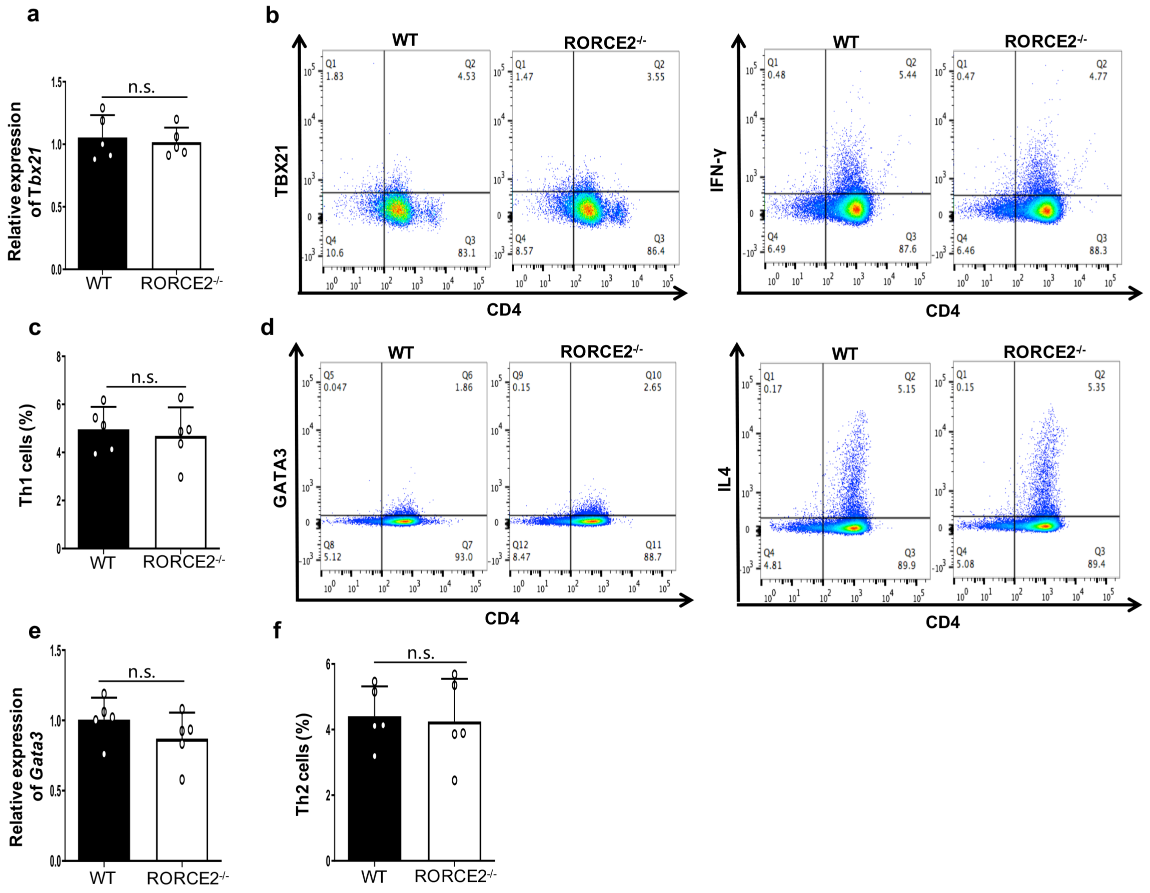

Supplementary Figure 5. RORCE2 deficiency has no effects on Th1 or Th2 cells *in vivo*.

a-c. Relative expression of *Tbx21* gene (a) and frequency of Th1 cells stained with an anti-TBX21 or anti-IFNγ mAb in RORCE2$^{-/-}$ and WT mice (b and c). d-f. Relative expression of *Gata3* gene (d) and frequency of Th2 cells stained with an anti-GATA3 or anti-IL-4 mAb in RORCE2$^{-/-}$ and WT mice (e and f). Mean ± SEM are shown, n=5 independent experiments, unpaired two-tailed Student's t-test (a, c, e, f). Experimental mice were between 8 and 12 weeks of age, with no preference to gender and were maintained on a C57BL/6 background. Source data are provided as a Source Data file.

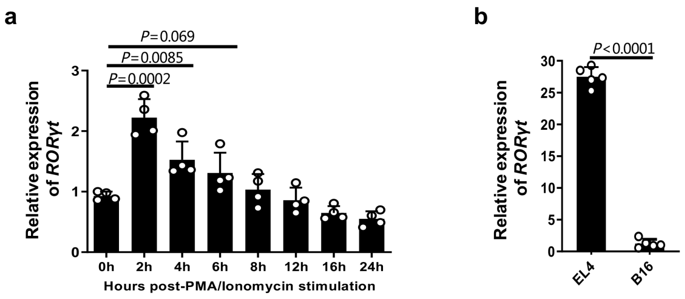

Supplementary Figure 6. The mRNA expression of *RORγt* in EL4 cells.

a. Relative mRNA expression levels of *RORγt (Rorc)* gene in PI-stimulated EL4 cells at indicated time points were measured by RT-qPCR. b. Relative mRNA expression levels of *RORγt (Rorc)* gene in EL4 and B16 cells after PI stimulation for 4 h were measured by RT-qPCR. Mean ± SEM are shown, n=4 or 5 (a with n=4 and b with n=5) independent experiments, unpaired two-tailed Student's t-test (a, b). Source data are provided as a Source Data file.

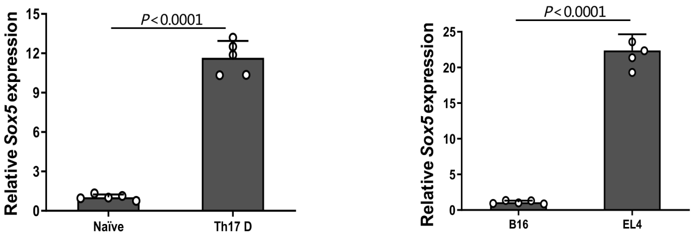

Supplementary Figure 7. Relative mRNA expression of the *Sox5* gene in Th17 cells.
Relative mRNA expression of *Sox5* gene in Th17-polarized and PI-stimulatedEL4 cells compared with that in naïve cells and PI-stimulated B16 cells, respectively. Th17 D represents Th17 differentiation under Th17 polarization conditions. Mean ± SEM are shown, n=5 independent experiments, unpaired two-tailed Student's t-test. Experimental mice were between 8 and 12 weeks of age, with no preference to gender and were maintained on a C57BL/6 background. Source data are provided as a Source Data file.

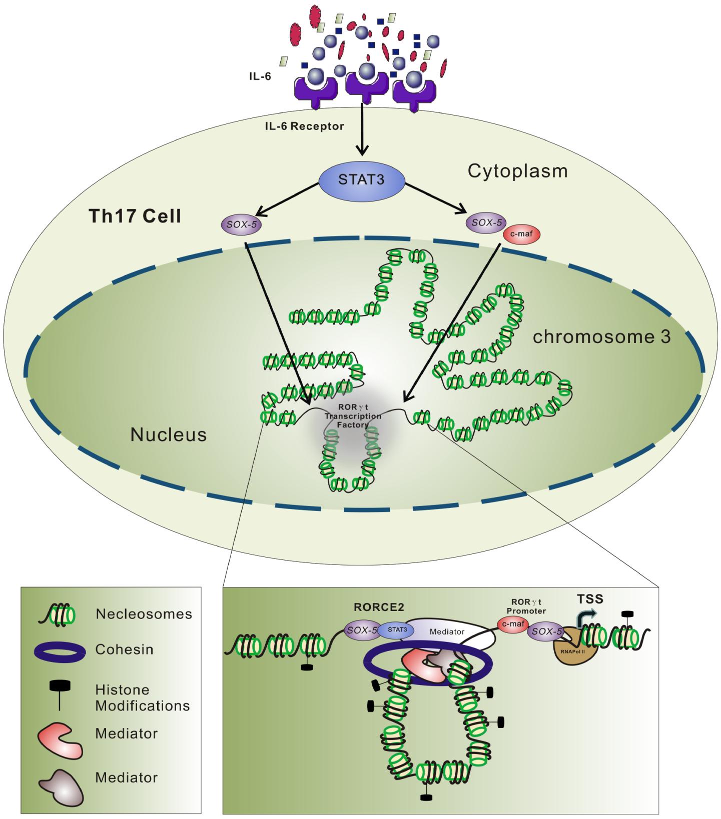

Supplementary Figure 8. The proposed model for RORCE2-mediated regulation of RORγt expression in Th17 cells.

Previous studies have indicated that SOX-5, as a downstream target of STAT3, and c-Maf cooperatively induce the induction of RORγt and differentiation of Th17 cells via binding to the RORγt promoter. In this study, we uncovered that *RORγ*t gene expression in Th17 cells was also mediated by a novel enhancer, named RORCE2. SOX-5 was required for chromatin looping between RORCE2 and RORγt promoter and for the binding of STAT3 to RORCE2, which is crucial for RORγt expression, Th17 cell differentiation and Th17-related diseases and most likely functions together with other TFs.

Supplementary Table 1. Primers used for PCR of top 10 potential off targets of upstream sgRNA or downstream sgRNA used in RORCE2$^{-/-}$mice.

Target	Primer name	Primer sequence (5´ to 3´)
**#1 potential off target of upstream sgRNAs**	SgRNAs-up-1_F	TCTCCCAGAGCACCCGAGTT
	SgRNAs-up-1_R	GTCTGTGTCAGAACGGGAGTG
**#2 potential off target of upstream sgRNAs**	SgRNAs-up-2_F	AGCCTCCCCAATGAAACGCAATG
	SgRNAs-up-2_R	CAATACACCTCTGAATGTCTCCCG
**#3 potential off target of upstream sgRNAs**	SgRNAs-up-3_F	CAGTTCCCAGCATCCATACAGG
	SgRNAs-up-3_R	GGCCATCCCTACCTTCTGGTAA
**#4 potential off target of upstream sgRNAs**	SgRNAs-up-4_F	GACTCAGTTTCAAACCTGGGCC
	SgRNAs-up-4_R	GTTGGACGTACCACAGCTTTCATC
**#5 potential off target of upstream sgRNAs**	SgRNAs-up-5_F	GGTTAGCACCGTTTAACCAGTGAG
	SgRNAs-up-5_R	CTCCTAGACTCCTCCTACCAC
**#6 potential off target of upstream sgRNAs**	SgRNAs-up-6_F	CCTCCTCGTCACACTAAACTCC
	SgRNAs-up-6_R	GAGCAGAATAGACACAAGCCCTCC
**#7 potential off target of upstream sgRNAs**	SgRNAs-up-7_F	CTGTAGGTGCACTTATCCTTCAGC
	SgRNAs-up-7_R	CAGTACCTCAACTCACCACCC
**#8 potential off target of upstream sgRNAs**	SgRNAs-up-8_F	TCTGGTCAGGATAAGCACTGAGC
	SgRNAs-up-8_R	GCTACGTGCTAAGGACCTACTATG
**#9 potential off target of upstream sgRNAs**	SgRNAs-up-9_F	CCAAGGACCAAGGCAAGAGAAG
	SgRNAs-up-9_R	CCCTAGCTTCCTCCTACCAG
**#10 potential off target of upstream sgRNAs**	SgRNAs-up-10_F	GCAGAATGAGCCAACTATGCCC
	SgRNAs-up-10_R	CCACAGTCCAGAGCAGAAGAAG
**#1 potential off target of downstream sgRNAs**	SgRNAs-down-1_F	ATTCCCTCACAAGCTGCTGAATCC
	SgRNAs-down-1_R	AACCTCTCACCCTGTGTGACATAG
**#2 potential off target of downstream sgRNAs**	SgRNAs-down-2_F	TCCAGTGCTTTCTTCAGGTAGCTC
	SgRNAs-down-2_R	AGCCTACTGTGCGAATGGTCTC
**#3 potential off target of downstream sgRNAs**	SgRNAs-down-3_F	CTGGTCCTAGTTCCCACCTC
	SgRNAs-down-3_R	CACCAACCCTGGGTTGAAATCC
**#4 potential off target of downstream sgRNAs**	SgRNAs-down-4_F	AAAGCTGATCTTTCGGATCCTGGG
	SgRNAs-down-4_R	CCCATCCAAGTCTTCTCTCAGTG
**#5 potential off target of downstream sgRNAs**	SgRNAs-down-5_F	GGTGATGAGTAAGGACTCTTCAGC
	SgRNAs-down-5_R	CTCGGTGCTGGGATTAAAGGC
**#6 potential off target of downstream sgRNAs**	SgRNAs-down-6_F	GGCACATCAGGATCACTCCTTC
	SgRNAs-down-6_R	CTGGGTTTGGTCTATGGCTATCTG
**#7 potential off target of downstream sgRNAs**	SgRNAs-down-7_F	ACCTGTTCCTCCCCCTATTTCAGA
	SgRNAs-down-7_R	GTCTCTCTGGGTGAGAGTGAAAG
**#8 potential off target of downstream sgRNAs**	SgRNAs-down-8_F	GAAAAGAAGGGATCAGGGAAGGG
	SgRNAs-down-8_R	GGAGATTGTAGTGCAGCCAACTTC
**#9 potential off target of downstream sgRNAs**	SgRNAs-down-9_F	GGGCTATGGGGTTTTGGATCTC
	SgRNAs-down-9_R	GACACAGATACACTGAGACAGTGC
**#10 potential off target of downstream sgRNAs**	SgRNAs-down-10_F	GTTCAGTGTCCAGTTTAAGGCTCC
	SgRNAs-down-10_R	AGTAGCCCCCTGTAGCTTCC

Supplementary Table 2. Primers used for reporter construct cloning.

Gene name	Primer name	Primer sequence (5´ to 3´)
RORCE1	RORCE1_F	5-TACGACGCGTTCAGTCTTCCACATCAAGTAATTGCAAT-3
	RORCE1_R	5-CTGAAGATCTCTAATGTTTGAAGCTGTCCATCCTACC-3
RORCE2	RORCE2_F	5-TACGACGCGT TTCCTCCTGGCAACTGCCTCTAAG-3
	RORCE2_R	5-CTGAAGATCTACAAGGAAGACTTTAACAGTGTGGTTTG-3
RORCE3	RORCE3_F	5-TACGACGCGTGAACTCCTCCTGCTCTTCCAGCCTCT-3
	RORCE3_R	5-CTGAAGATCTAGTTACAGAGACAAAATTTGGAGCTGTG-3
RORCE1/2	RORCE1/2_F	5-TACGACGCGTTCAGTCTTCCACATCAAGTAATTGCAAT-3
	RORCE1/2_R	5-CTGAAGATCTACAAGGAAGACTTTAACAGTGTGGTTTG-3
RORCE2/3	RORCE2/3_F	5-TACGACGCGT TTCCTCCTGGCAACTGCCTCTAAG-3
	RORCE2/3_R	5-CTGAAGATCTAGTTACAGAGACAAAATTTGGAGCTGTG-3
RORCEf	RORCEf_F	5-TACGACGCGTTCAGTCTTCCACATCAAGTAATTGCAAT-3
	RORCEf_R	5-CTGAAGATCTAGTTACAGAGACAAAATTTGGAGCTGTG-3

Supplementary Table 3. Primers used for ChIP-qPCR.

Gene name	Primer name	Primer sequence (5′ to 3′)
Primer A	Primer A_F	5-CCACACAGTGCCCTTTAACC-3
	Primer A_R	5-ATGTCCCAGATCCCAGTCC-3
Primer B	Primer B _F	5-CAGCCAGAAGATGAAGGTG-3
	Primer B _R	5-GCTAAGAGGAAGGAGGAAGTG-3
Primer C	Primer C_F	5-AACCTGGCACTTCGCACTTA-3
	Primer C_R	5-GCATTGCTCACTAGGGATTG-3
Primer D	Primer D_F	5-ATTGGCTTTGGCTGTGAGG-3
	Primer D_R	5-CTACTGTGCTAATGGCGGT-3
Primer E	Primer E_F	5-CCACGTTATGAGGTGCTGTAGG-3
	Primer E_R	5-TGGAGGAGTAGCGGGAAAGA-3
Primer F	Primer F_F	5-CGAGTTGAAGTCTCCGTAAGC-3
	Primer F_R	5-TCCCAAGGCAGCAGGTAA-3
SOX-5-BS	SOX-5 BS_F	5-TCCACTATGTTCCCACCAC-3
	SOX-5 BS_R	5-GTCAGCACGGAGGATTGTT-3
RORγt p	RORγt_F	5-TTGACAGTCCACAGGGTCTC-3
	RORγt _R	5-GCCCTCTTCACCAAGTGACA-3
STAT3-BS	STAT3 BS_F	5-GGGTAGGATGGACAGCTTCA-3
	STAT3 BS_R	5-AACAACTGTACACTCACCTTAG-3

Supplementary Table 4. Primers used for RT-qPCR.

Gene name	Primer name	Primer sequence (5´ to 3´)
RORγt	RORγt _F	5-CGAGATGCTGTCAAGTTTGG-3
	RORγt _R	5-CACTTGTTCCTGTTGCTGCT-3
IL-17A	IL-17A _F	5-TCCAGAAGGCCCTCAGACTA-3
	IL-17A _R	5-TCAGGACCAGGATCTCTTGC-3
Tbx21	Tbx21_F	5-GCCAGGGAACCGGTTATATG-3
	Tbx21 _R	5-GACGATCATCTGGGTCACAT-3
Gata3	Gata3_F	5-AAGCTCAGTATCCGCTGACG-3
	Gata3_R	5-GTTTCCGTAGTAGGACGGGAC-3
Sox5	Sox5_F	5-ACATCGGGAAGTAGGAGAGACTGA-3
	Sox5_R	5-TACCTCTCCATCTGTCTCCCCATA-3
Stat3	Stat3_F	5-CTAACATTCTGGGCACGAAC-3
	Stat3-R	5- AAACTGGCAAGGAGTGGGTC-3
GAPDH	GAPDH _F	5-ACAGCCGCATCTTCTTGTGCAGTG-3
	GAPDH _R	5-GGCCTTGACTGTGCCGTTGAATTT-3

Supplementary Table 5. Primers used for 3C-qPCR and ChIP-loop assays.

**Gene name**	**Primer name**	**Primer sequence (5′ to 3′)**
GAPDH	GAPDH_F_3C	5-TACCTGATGAACCTAAGC-3
	GAPDH_R_3C	5-GTGAAAGGGGCAGTGTCT-3
NCS	NCS	5-GTCTCAAAATAACACCCCAC-3
TS1	TS1	5-ATCAAGAGAATCCCCCACT-3
TS2	TS2	5-CACTTCCTCCTTCCTCTT-3
TS3	TS3	5-TCCTGACAACTGCCTCTAA-3
TS4	TS4	5-CTTCGCCCTAAGACAGATA-3
TS5	TS5	5-AGGGAGGCAAAATTACAG-3
TS6	TS6	5-GTTCAGTCACATTCCCAC-3
TS7	TS7	5-TACCGCCATTAGCACAGT-3
TS8	TS8	5-AGAGGGTCCAGTGAAAGAT-3
AS	AS	5-TTCCAAAACCACAGCTAC-3
