# Supplementary material for: SOX-5 activates a novel RORγt enhancer to facilitate experimental autoimmune encephalomyelitis by promoting Th17 cell differentiation
Source: Nat Commun. 2021 Jan 20;12:481. doi: 10.1038/s41467-020-20786-w (PMC7817841; doi:10.1038/s41467-020-20786-w)
Supplement: Supplementary file 3 — Reporting Summary [file 41467_2020_20786_MOESM3_ESM.pdf]

nature research

Corresponding author(s): Yuzhang Wu (NCOMMS-20-02627)

Last updated by author(s): Dec 10, 2020

Reporting Summary

Nature Research wishes to improve the reproducibility of the work that we publish. This form provides structure for consistency and transparency in reporting. For further information on Nature Research policies, see Authors & Referees and the Editorial Policy Checklist.

Statistics

For all statistical analyses, confirm that the following items are present in the figure legend, table legend, main text, or Methods section.

n/a	Confirmed	
☐	☒	The exact sample size (*n*) for each experimental group/condition, given as a discrete number and unit of measurement
☐	☒	A statement on whether measurements were taken from distinct samples or whether the same sample was measured repeatedly
☐	☒	The statistical test(s) used AND whether they are one- or two-sided<br>*Only common tests should be described solely by name; describe more complex techniques in the Methods section.*
☒	☐	A description of all covariates tested
☒	☐	A description of any assumptions or corrections, such as tests of normality and adjustment for multiple comparisons
☐	☒	A full description of the statistical parameters including central tendency (e.g. means) or other basic estimates (e.g. regression coefficient) AND variation (e.g. standard deviation) or associated estimates of uncertainty (e.g. confidence intervals)
☐	☒	For null hypothesis testing, the test statistic (e.g. *F*, *t*, *r*) with confidence intervals, effect sizes, degrees of freedom and *P* value noted<br>*Give P values as exact values whenever suitable.*
☒	☐	For Bayesian analysis, information on the choice of priors and Markov chain Monte Carlo settings
☒	☐	For hierarchical and complex designs, identification of the appropriate level for tests and full reporting of outcomes
☒	☐	Estimates of effect sizes (e.g. Cohen's *d*, Pearson's *r*), indicating how they were calculated

Our web collection on statistics for biologists contains articles on many of the points above.

Software and code

Policy information about availability of computer code

Data collection	Data collection of flow cytometry experiments was performed on BD FACS Canto II (BD,USA) and BD FACS ARIA II (BD,USA) instruments. Data collection of qPCR experiments was performed on BIO-RAD C1000 Touch (BIO-RAD,USA) and CFX96TM Real-Time System (BIO-RAD,USA). Images of tissue section staining experiments were performed on M8 Digital scanning microimaging system (Precipoint, Germany). Images of WB were performed on Fusion solo-s (Vilber, France). Data collection of dual-luciferase experiments were performed on GloMax Multi Jr(Promega, USA).
Data analysis	FlowJo (10.0.7) was used for flow cytometry data analysis. The bowtie (2.3.5), SICER and IGV (2.8.13) were used for ChIP-seq data analysis. Viewpoint (1) was used for images analysis of tissue section staining experiments.For additional details please refer to Methods section. GraphPad Prism (8) was used for the statistical test.

For manuscripts utilizing custom algorithms or software that are central to the research but not yet described in published literature, software must be made available to editors/reviewers. We strongly encourage code deposition in a community repository (e.g. GitHub). See the Nature Research guidelines for submitting code & software for further information.

Data

Policy information about availability of data

All manuscripts must include a data availability statement. This statement should provide the following information, where applicable:
- Accession codes, unique identifiers, or web links for publicly available datasets
- A list of figures that have associated raw data
- A description of any restrictions on data availability

We downloaded the mouse H3K4me2 ChIP-seq data, including GSM1032374, 1032375, 2258669, 2258675 and 2258681, from the GEO database. We also used the Roadmap Epigenomics Project database (http://egg2.wustl.edu/roadmap/web_portal/) to analyze the chromatin signature of a region similar to mouse RORCE (indicated by the red box) including H3K27me3, H3K9me3, H3K27Ac, H3K36me3, H3K4me1 and H3K4me3 in human Th17 and Th0 cells. We also used the JASPAR database to predict the transcriptional factor binding sites in RORCE (http://jaspar.genereg.net/). All the other data supporting the findings of this study are

available within the article and its supplementary information files or available from the corresponding author upon reasonable request. The source data underlying Figures and Supplementary Figures are provided as a Source Data file.

Field-specific reporting

Please select the one below that is the best fit for your research. If you are not sure, read the appropriate sections before making your selection.

☒ Life sciences ☐ Behavioural & social sciences ☐ Ecological, evolutionary & environmental sciences

For a reference copy of the document with all sections, see nature.com/documents/nr-reporting-summary-flat.pdf

Life sciences study design

All studies must disclose on these points even when the disclosure is negative.

Sample size	No statistical methods were used to predetermine sample size. The samples sizes were chosen based on previous studies with similar methodologies. The references of cellular experiments sample size are: Yahia-Cherbal H, et al. NFAT primes the human RORC locus for RORgammat expression in CD4(+) T cells. Nat Commun 10, 4698 (2019); Salem T, et al. Chromatin loop organization of the junb locus in mouse dendritic cells. Nucleic Acids Res 41, 8908-8925 (2013). The references of in vivo experiments are: Kim HS, et al. PTEN drives Th17 cell differentiation by preventing IL-2 production. J Exp Med 214, 3381-3398 (2017); Tanaka S, et al. Sox5 and c-Maf cooperatively induce Th17 cell differentiation via RORgammat induction as downstream targets of Stat3. J Exp Med 211, 1857-1874 (2014). Additional details for each figure panel are included in the figure legends.
Data exclusions	No data exclusion were performed.
Replication	Experiments were replicated at least twice as described throughout the manuscript and in the Methods. Additional information of the each figure panel is included in the figure legend.
Randomization	We did not use any randomization. All mice of same background, similar age and sex were used in all experiments.
Blinding	The investigators were not blinded to the identities of the samples because treatments and data collection were performed by the same people. However, data analysis were processed by other authors who were blinded to the group allocation.

Behavioural & social sciences study design

All studies must disclose on these points even when the disclosure is negative.

Study description	*Briefly describe the study type including whether data are quantitative, qualitative, or mixed-methods (e.g. qualitative cross-sectional, quantitative experimental, mixed-methods case study).*
Research sample	*State the research sample (e.g. Harvard university undergraduates, villagers in rural India) and provide relevant demographic information (e.g. age, sex) and indicate whether the sample is representative. Provide a rationale for the study sample chosen. For studies involving existing datasets, please describe the dataset and source.*
Sampling strategy	*Describe the sampling procedure (e.g. random, snowball, stratified, convenience). Describe the statistical methods that were used to predetermine sample size OR if no sample-size calculation was performed, describe how sample sizes were chosen and provide a rationale for why these sample sizes are sufficient. For qualitative data, please indicate whether data saturation was considered, and what criteria were used to decide that no further sampling was needed.*
Data collection	*Provide details about the data collection procedure, including the instruments or devices used to record the data (e.g. pen and paper, computer, eye tracker, video or audio equipment) whether anyone was present besides the participant(s) and the researcher, and whether the researcher was blind to experimental condition and/or the study hypothesis during data collection.*
Timing	*Indicate the start and stop dates of data collection. If there is a gap between collection periods, state the dates for each sample cohort.*
Data exclusions	*If no data were excluded from the analyses, state so OR if data were excluded, provide the exact number of exclusions and the rationale behind them, indicating whether exclusion criteria were pre-established.*
Non-participation	*State how many participants dropped out/declined participation and the reason(s) given OR provide response rate OR state that no participants dropped out/declined participation.*
Randomization	*If participants were not allocated into experimental groups, state so OR describe how participants were allocated to groups, and if allocation was not random, describe how covariates were controlled.*

Ecological, evolutionary & environmental sciences study design

All studies must disclose on these points even when the disclosure is negative.

Study description	*Briefly describe the study. For quantitative data include treatment factors and interactions, design structure (e.g. factorial, nested, hierarchical), nature and number of experimental units and replicates.*
Research sample	*Describe the research sample (e.g. a group of tagged Passer domesticus, all Stenocereus thurberi within Organ Pipe Cactus National Monument), and provide a rationale for the sample choice. When relevant, describe the organism taxa, source, sex, age range and any manipulations. State what population the sample is meant to represent when applicable. For studies involving existing datasets, describe the data and its source.*
Sampling strategy	*Note the sampling procedure. Describe the statistical methods that were used to predetermine sample size OR if no sample-size calculation was performed, describe how sample sizes were chosen and provide a rationale for why these sample sizes are sufficient.*
Data collection	*Describe the data collection procedure, including who recorded the data and how.*
Timing and spatial scale	*Indicate the start and stop dates of data collection, noting the frequency and periodicity of sampling and providing a rationale for these choices. If there is a gap between collection periods, state the dates for each sample cohort. Specify the spatial scale from which the data are taken*
Data exclusions	*If no data were excluded from the analyses, state so OR if data were excluded, describe the exclusions and the rationale behind them, indicating whether exclusion criteria were pre-established.*
Reproducibility	*Describe the measures taken to verify the reproducibility of experimental findings. For each experiment, note whether any attempts to repeat the experiment failed OR state that all attempts to repeat the experiment were successful.*
Randomization	*Describe how samples/organisms/participants were allocated into groups. If allocation was not random, describe how covariates were controlled. If this is not relevant to your study, explain why.*
Blinding	*Describe the extent of blinding used during data acquisition and analysis. If blinding was not possible, describe why OR explain why blinding was not relevant to your study.*

Did the study involve field work? ☐ Yes ☐ No

Field work, collection and transport

Field conditions	*Describe the study conditions for field work, providing relevant parameters (e.g. temperature, rainfall).*
Location	*State the location of the sampling or experiment, providing relevant parameters (e.g. latitude and longitude, elevation, water depth).*
Access and import/export	*Describe the efforts you have made to access habitats and to collect and import/export your samples in a responsible manner and in compliance with local, national and international laws, noting any permits that were obtained (give the name of the issuing authority, the date of issue, and any identifying information).*
Disturbance	*Describe any disturbance caused by the study and how it was minimized.*

Reporting for specific materials, systems and methods

We require information from authors about some types of materials, experimental systems and methods used in many studies. Here, indicate whether each material, system or method listed is relevant to your study. If you are not sure if a list item applies to your research, read the appropriate section before selecting a response.

Materials & experimental systems

n/a	Involved in the study	
☐	☒	Antibodies
☐	☒	Eukaryotic cell lines
☒	☐	Palaeontology
☐	☒	Animals and other organisms
☒	☐	Human research participants
☒	☐	Clinical data

Methods

n/a	Involved in the study	
☒	☐	ChIP-seq
☐	☒	Flow cytometry
☒	☐	MRI-based neuroimaging

Antibodies

Antibodies used

Antibody	Supplier	Catalog number	Clone(species)	Dilution or Final concentration
Anti-CD3	eBioscience	17-0031-63	145-2C11(Mouse, Human)	1:100 for flow cytometry
Anti-CD4	eBioscience	45-0049-42	RPA-T4(Mouse, Human)	1:100 for flow cytometry
Anti-CD8	eBioscience	17-0081-82	53-6.7(Mouse)	1:100 for flow cytometry
Anti-CD19	eBioscience	RM7705	6D5(Mouse)	1:100 for flow cytometry
Anti-CD45	eBioscience	45-0451-82	30-F11(Mouse, Human)	1:100 for flow cytometry
Anti-CD127	eBioscience	48-1271-82	A7R34(Mouse)	1:100 for flow cytometry
Anti-Gr-1	eBioscience	17-5931-82	RB6-8C5(Mouse, Dog, Fish, Human)	1:100 for flow cytometry
Anti-RORγt	eBioscience	17-6988-82	AFKJS-9(Mouse, Human)	1:100 for flow cytometry
Anti-IL-17	eBioscience	12-7177-81	eBio17B7 (Human, Mouse, Rat)	1:100 for flow cytometry
Anti-TBX21	eBioscience	17-5825-82	eBio4B10(Mouse, Human)	1:100 for flow cytometry
Anti-IFN-γ	eBioscience	12-7311-82	XMG1.2(Mouse, Human)	1:100 for flow cytometry
Anti-GATA3	eBioscience	50-9966-42	TWAJ(Mouse, Human)	1:100 for flow cytometry
Anti-IL-4	eBioscience	12-7041-82	11B11(Mouse, Rat)	1:100 for flow cytometry
Anti-SOX-5	Abcam	ab94396	polyclone(Mouse, Human)	1:250 for ChIP or 1:1000 for WB
Anti-STAT3	Cell Signaling Technology	9139	124H6(Mouse, Human, Rat, Monkey)	1:250 for ChIP/IP or 1:1000 for WB
Anti-H3K4me1	Abcam	ab176877	ERP16597(Mouse, Rat, Human)	1:250 for ChIP
Anti-H3K27ac	Abcam	ab4729	Polyclone(Mouse, Cow, Human)	1:250 for ChIP
Anti-HA	Abcam	ab18181	HA.C5(all)	1:250 for IP or 1:1000 for WB
Anti-Flag	Sigma-Aldrich	F3165	M2(all)	1:1000 for WB
Anti-CD3	BioXCell	BP0001-1	145-2C11(Mouse)	2 μg/ml for T cell stimulation
Anti-CD28	BioXCell	BE0328	D665(Mouse)	2 μg/ml for T cell stimulation
Anti-IL-4	BioXCell	BP0045	11B11(Mouse)	10 μg/ml for T cell stimulation
Anti-IFNγ	BioXCell	BP0055	XMG1.2(mouse)	10 μg/ml for T cell stimulation

Validation

Antibodies were validated by the manufacturer and our experiments. Antibodies of anti-CD3, Anti-CD4,Anti-CD8, Anti-CD19, Anti-CD45, Anti-CD127, Anti-Gr-1, Anti-RORγt, Anti-IL-17, Anti-T-bet, Anti-IFN-γ, Anti-GATA3 and Anti-IL-4 from eBioscience were validated for flow cytometry of detecting mouse species proteins. Antibodies of Anti-SOX-5, Anti-STAT3, Anti-HA and Anti-Flag were validated for western blot of detecting mouse species proteins. Antibodies of Anti-SOX-5, Anti-STAT3, Anti-H3K4me1 and Anti-H3K27ac were validated for ChIP experiments of detecting mouse species proteins. Antibodies of Anti-STAT3 and Anti-HA were validated for IP experiments of detecting mouse species proteins. Antibodies of Anti-CD3, Anti-CD28, Anti-IL-4 and Anti-IFNγ from BioXCell were validated for mouse T cell activation.

Eukaryotic cell lines

Policy information about cell lines

Cell line source(s)

EL4, 293T, Hela and B16 cells were obtained from the American Type Culture Collection (ATCC).

Authentication

No further authentication was performed.

Mycoplasma contamination

The EL4, 293T, Hela and B16 cell lines were tested negative for mycoplasma contamination.

Commonly misidentified lines
(See ICLAC register)

No commonly misidentified cell lines were used in the study

Palaeontology

Specimen provenance

Provide provenance information for specimens and describe permits that were obtained for the work (including the name of the issuing authority, the date of issue, and any identifying information).

Specimen deposition

Indicate where the specimens have been deposited to permit free access by other researchers.

Dating methods

If new dates are provided, describe how they were obtained (e.g. collection, storage, sample pretreatment and measurement), where they were obtained (i.e. lab name), the calibration program and the protocol for quality assurance OR state that no new dates are provided.

☐ Tick this box to confirm that the raw and calibrated dates are available in the paper or in Supplementary Information.

Animals and other organisms

Policy information about studies involving animals; ARRIVE guidelines recommended for reporting animal research

Laboratory animals

C57BL/6 male and female mice were obtained from Beijing Biocytogen Co. RORCE2-deficient mice (C57BL/6 background) were generated by Beijing Biocytogen Co.The Sox5-BS-deficient mice (C57BL/6 background) were generated by Dr. Yiqiang Cui of Nanjing Medical University .All mice were housed in groups of 4-5 per cage in standard closed plastic cages containing bedding, enrichment, food, and water, at controlled stable room temperature and humidity, light/dark cycle 12 hours per day. All mice were maintained on a C57BL/6 background under specific pathogen-free conditions and bred in the animal facility of Institute of

	immunology of Third Military Medical University.8~12 weeks old mice were used in this study.
Wild animals	No wild animals were used.
Field-collected samples	No field-collected samples were used.
Ethics oversight	All mouse experiments were performed in accordance with the guidelines of the Institutional Animal Care and Use Committees of the Third Military Medical University.

Note that full information on the approval of the study protocol must also be provided in the manuscript.

Human research participants

Policy information about studies involving human research participants

Population characteristics	*Describe the covariate-relevant population characteristics of the human research participants (e.g. age, gender, genotypic information, past and current diagnosis and treatment categories). If you filled out the behavioural & social sciences study design questions and have nothing to add here, write "See above."*
Recruitment	*Describe how participants were recruited. Outline any potential self-selection bias or other biases that may be present and how these are likely to impact results.*
Ethics oversight	*Identify the organization(s) that approved the study protocol.*

Note that full information on the approval of the study protocol must also be provided in the manuscript.

Clinical data

Policy information about clinical studies

All manuscripts should comply with the ICMJE guidelines for publication of clinical research and a completed CONSORT checklist must be included with all submissions.

Clinical trial registration	*Provide the trial registration number from ClinicalTrials.gov or an equivalent agency.*
Study protocol	*Note where the full trial protocol can be accessed OR if not available, explain why.*
Data collection	*Describe the settings and locales of data collection, noting the time periods of recruitment and data collection.*
Outcomes	*Describe how you pre-defined primary and secondary outcome measures and how you assessed these measures.*

ChIP-seq

Data deposition

☐ Confirm that both raw and final processed data have been deposited in a public database such as GEO.

☐ Confirm that you have deposited or provided access to graph files (e.g. BED files) for the called peaks.

Data access links<br>*May remain private before publication.*	*For "Initial submission" or "Revised version" documents, provide reviewer access links. For your "Final submission" document, provide a link to the deposited data.*
Files in database submission	*Provide a list of all files available in the database submission.*
Genome browser session<br>(e.g. UCSC)	*Provide a link to an anonymized genome browser session for "Initial submission" and "Revised version" documents only, to enable peer review. Write "no longer applicable" for "Final submission" documents.*

Methodology

Replicates	*Describe the experimental replicates, specifying number, type and replicate agreement.*
Sequencing depth	*Describe the sequencing depth for each experiment, providing the total number of reads, uniquely mapped reads, length of reads and whether they were paired- or single-end.*
Antibodies	*Describe the antibodies used for the ChIP-seq experiments; as applicable, provide supplier name, catalog number, clone name, and lot number.*
Peak calling parameters	*Specify the command line program and parameters used for read mapping and peak calling, including the ChIP, control and index files used.*
Data quality	*Describe the methods used to ensure data quality in full detail, including how many peaks are at FDR 5% and above 5-fold enrichment.*

Software | Describe the software used to collect and analyze the ChIP-seq data. For custom code that has been deposited into a community repository, provide accession details.

Flow Cytometry

Plots

Confirm that:

☒ The axis labels state the marker and fluorochrome used (e.g. CD4-FITC).

☒ The axis scales are clearly visible. Include numbers along axes only for bottom left plot of group (a 'group' is an analysis of identical markers).

☒ All plots are contour plots with outliers or pseudocolor plots.

☒ A numerical value for number of cells or percentage (with statistics) is provided.

Methodology

Sample preparation

(1) The isolation of analysis of splenic CD4+ T cells: To prepare single-cell suspensions, the spleens of mice were mechanically dissociated and passed through 70-µm mesh (BD Biosciences). CD4+ T cells were purified using an EasySep™ mouse CD4+ T cell isolation kit according to the manufacturer's instructions (STEMCELL Technologies). The purifed cells were then stimulated with PMA (50 ng/ml, Sigma-Aldrich), ionomycin (1 uM, Calbiochem)for 2 hours. Then, we add the protein transport inhibitor Golgi-Stop (BD Bioscience) at a final concentration of 3 uM in the last 2 hours of stimulation and then stained with anti-CD4, anti-IFN-γ, anti-IL4, anti-IL17A, anti-TBX21, anti-GATA3 and anti-RORγt antibodies for flow cytometry analysis (all antibodies were purchased from eBioscience).

(2) Sorting CD4+IL17+ Th17 cells and CD4+IL17- T cells: To prepare single-cell suspensions, the spleen and mesenteric lymph nodes of IL17-IRES-EGFP mice (purchased from Beijing Biocytogen Co., Ltd) were mechanically dissociated and passed through 70-µm mesh (BD Biosciences). CD4+ T cells were purified using an EasySep™ mouse CD4+ T cell isolation kit according to the manufacturer's instructions (STEMCELL Technologies) and then incubated for 30 min at room temperature with an anti-mCD4 antibody (PerCP-Cy5.5A). Subsequently, the CD4+ T cells were sorted into CD4+IL17+ Th17 cells (from an IL17-EGFP mouse) and CD4+IL17- T cells based on CD4 and GFP expression analyzed by FACS, and the sorting purity was retested with anti-CD4 and anti-RORγt monoclonal antibodies (mAbs). All antibodies were purchased from eBioscience.

(3) Differentiation of CD4+ T cells in vitro: Naïve CD4+ T cells were isolated from 8- to 12-week-old mice with an EasySep™ mouse naïve CD4+ T cell isolation kit (STEMCELL) according to the manufacturer's instructions. Sorted naïve CD4+ T cells were cultured on irradiated splenocytes (2000 rads) with soluble anti-CD3 (2 µg/ml, 145-2C11; BioXCell) at a ratio of 1:5 in a 24-well . The naïve cells were cultured at 1.5×10^6/ml in T cell medium, sodium pyruvate, Hepes, penicillin/streptomycin, gentamicin sulfate, and 2-mercaptoethanol. The following cytokines were added to generate Th17 subset: IL-6 (20 ng/ml; Miltenyi Biotec), TGF-β1 (2 ng/ml; Miltenyi Biotec), anti- IL-4 (10 ug/ml; 11B11; BioXCell) and anti-IFNγ(10 ug/ml; XMG1.2; BioXCell). On day 3 after stimulation, we transferred 4 24-wells into 1 10 cm-dish containing 10 ml of T cell medium, IL-6 (20 ng/ml; Miltenyi Biotec), TGF-β1 (2 ng/ml; Miltenyi Biotec), anti- IL-4 (10 ug/ml; 11B11; BioXCell) and anti-IFNγ(10 ug/ml; XMG1.2; BioXCell). This time we supplemented IL-2 (15 U/ml) into the T cell media and cultured the cells for additional 2 days before analysis. Th1 and Th2 polarizations were performed using CellXVivo mouse Th1 cell differentiation kit (R&D Systems,CDK018) and CellXVivo mouse Th2 cell differentiation kit (R&D Systems,CDK019), respectively.The polarized cells were then stimulated with PMA (50 ng/ml, Sigma-Aldrich), ionomycin (1 uM, Calbiochem)for 2 hours. Then, we add the protein transport inhibitor Golgi-Stop (BD Bioscience) at a final concentration of 3 uM in the last 2 hours of stimulation and then stained with anti-CD4, anti-IFN-γ, anti-IL4,anti-IL17 and anti-RORγt antibodies for flow cytometry analysis (all antibodies were purchased from eBioscience).

(4) Isolation and analysis of spinal cord mononuclear cells: Mice were anesthetized and perfused with cold PBS. The spinal cord was then removed, cut into 0.5-cm pieces, digested with the Neural Tissue Dissociation Kit (Miltenyi Biotec), and homogenized. The mononuclear cells in the spinal cord were isolated by gradient centrifugation at 850 g for 30 min on a 40%/80% Percoll gradient. The isolated cells were then stimulated with PMA (50 ng/ml, Sigma-Aldrich), ionomycin (1 uM, Calbiochem)for 2 hours. Then, we add the protein transport inhibitor Golgi-Stop (BD Bioscience) at a final concentration of 3 uM in the last 2 hours of stimulation and then stained with anti-CD45, anti-CD3, anti-IFN-γ, anti-CD4, anti-IL4, anti-IL17 and anti-RORγt antibodies for flow cytometry analysis (all antibodies were purchased from eBioscience).

(5) Isolation and analysis of lamina propria lymphocytes: The pieces of tissue were digested for 1 h at 37°C in a digestion solution containing 4% FBS, 0.5 mg/ml collagenase III (Roche), 0.2 mg/ml DNase I (Sigma-Aldrich), and 2 mg/ml dispase II (Sigma-Aldrich) after removing the epithelial cell layer, and lymphocytes were obtained by gradient centrifugation on a 40%/80% Percoll gradient (GE Healthcare). For flow cytometry analysis, isolated lymphocytes were stained with an anti-lineage cocktail (including anti-CD19, anti-CD8, anti-Gr1, and anti-CD3 antibodies) and anti-CD45, anti-CD127, anti-CD4, anti-IL17 and anti-RORγt antibodies (all antibodies were purchased from eBioscience).

For surface staining of all the flow cytometry, cells were stained with the appropriate antibodies for 30 min at 4°C. For intracellular staining, cells were fixed and permeabilized with Perm/Fix (eBioscience), washed two times with Perm/Wash (eBioscience) and then stained with appropriate antibodies for 30 min in PBS containing 2% FBS.

Instrument | BD FACSCanto II,BD FACS ARIA II

Software | BD FACSCanto cell analyzer,FlowJo 10.0.07

Cell population abundance | Cell subsets were sorted on a BD FACS Aria II instrument. Post sort samples were analyzed on the same FACS Aria III machine indicating greater than 90% purity for sorted subsets.

Gating strategy | (1)For analysis of Th1, Th2 or Th17 cells in the spleen of mice, cells were gated on lymphocytes in FCS/SSC gate, followed by exclusion of doublets in FSC-A/FSC-H parameter.
(2)For analysis of protein expression in Th17 cells, cells were gated on lymphocytes in FCS/SSC gate, followed by exclusion of

doublets in FSC-A/FSC-H parameter, followed gating by CD4+RORγt+.
(3)For sorting CD4+IL17+ Th17 cells in the spleen of IL-17-IRES-EGFP reporter mice, cells were gated on lymphocytes in FCS/SSC gate, followed by exclusion of doublets in FSC-A/FSC-H parameter, followed by gating on CD4+GFP+;
(4)For analysis of polarized Th1,Th2 or Th17 cells, cells were gated on lymphocytes in FCS/SSC gate, followed by exclusion of doublets in FSC-A/FSC-H parameter.
(5)For analysis of ILC3 in the lamina propria lymphocytes, cells were gated on lymphocytes in FCS/SSC gate, followed by exclusion of doublets in FSC-A/FSC-H parameter, followed by gating on CD45+Lin-.
(6)For sorting of Lin-CD45+CD127+ in small intestines and colons, cells were gated on lymphocytes in FCS/SSC gate, followed by exclusion of doublets in FSC-A/FSC-H parameter, followed by gating on Lin-CD45+CD127+.
(7)For analysis of Th17 in the lamina propria lymphocytes, cells were gated on lymphocytes in FCS/SSC gate, followed by exclusion of doublets in FSC-A/FSC-H parameter, followed by gating on Lin+CD45+CD4+.
(8)For analysis of Th17, Th1 or Th2 cells in the spinal cord mononuclear cells of EAE mice, cells were gated on single cells in FCS/SSC gate, followed by exclusion of doublets in FSC-A/FSC-H parameter. And then the living cells were followed by gating on CD45 +CD4+.
The boundaries between the positive and negative populations were defined based on isotype stainings.

☒ Tick this box to confirm that a figure exemplifying the gating strategy is provided in the Supplementary Information.

Magnetic resonance imaging

Experimental design

Design type — *Indicate task or resting state; event-related or block design.*

Design specifications — *Specify the number of blocks, trials or experimental units per session and/or subject, and specify the length of each trial or block (if trials are blocked) and interval between trials.*

Behavioral performance measures — *State number and/or type of variables recorded (e.g. correct button press, response time) and what statistics were used to establish that the subjects were performing the task as expected (e.g. mean, range, and/or standard deviation across subjects).*

Acquisition

Imaging type(s) — *Specify: functional, structural, diffusion, perfusion.*

Field strength — *Specify in Tesla*

Sequence & imaging parameters — *Specify the pulse sequence type (gradient echo, spin echo, etc.), imaging type (EPI, spiral, etc.), field of view, matrix size, slice thickness, orientation and TE/TR/flip angle.*

Area of acquisition — *State whether a whole brain scan was used OR define the area of acquisition, describing how the region was determined.*

Diffusion MRI ☐ Used ☐ Not used

Preprocessing

Preprocessing software — *Provide detail on software version and revision number and on specific parameters (model/functions, brain extraction, segmentation, smoothing kernel size, etc.).*

Normalization — *If data were normalized/standardized, describe the approach(es): specify linear or non-linear and define image types used for transformation OR indicate that data were not normalized and explain rationale for lack of normalization.*

Normalization template — *Describe the template used for normalization/transformation, specifying subject space or group standardized space (e.g. original Talairach, MNI305, ICBM152) OR indicate that the data were not normalized.*

Noise and artifact removal — *Describe your procedure(s) for artifact and structured noise removal, specifying motion parameters, tissue signals and physiological signals (heart rate, respiration).*

Volume censoring — *Define your software and/or method and criteria for volume censoring, and state the extent of such censoring.*

Statistical modeling & inference

Model type and settings — *Specify type (mass univariate, multivariate, RSA, predictive, etc.) and describe essential details of the model at the first and second levels (e.g. fixed, random or mixed effects; drift or auto-correlation).*

Effect(s) tested — *Define precise effect in terms of the task or stimulus conditions instead of psychological concepts and indicate whether ANOVA or factorial designs were used.*

Specify type of analysis: ☐ Whole brain ☐ ROI-based ☐ Both

Statistic type for inference
(See Eklund et al. 2016) — *Specify voxel-wise or cluster-wise and report all relevant parameters for cluster-wise methods.*

Correction

Describe the type of correction and how it is obtained for multiple comparisons (e.g. FWE, FDR, permutation or Monte Carlo).

Models & analysis

n/a	Involved in the study
☐	☐ Functional and/or effective connectivity
☐	☐ Graph analysis
☐	☐ Multivariate modeling or predictive analysis

Functional and/or effective connectivity

Report the measures of dependence used and the model details (e.g. Pearson correlation, partial correlation, mutual information).

Graph analysis

Report the dependent variable and connectivity measure, specifying weighted graph or binarized graph, subject- or group-level, and the global and/or node summaries used (e.g. clustering coefficient, efficiency, etc.).

Multivariate modeling and predictive analysis

Specify independent variables, features extraction and dimension reduction, model, training and evaluation metrics.
